# Demand for the Reorganization of Veterinary Studies in Lower Austria

**Published:** 1897-05

**Authors:** 


Demand for the Reorganization of Veterinary Studies in Lower Austria. At the session of the Lower Austrian Landtag of January 21, 1896, a resolution was adopted urging the government to begin at once the reorganization of veterinary instruction, by means of which special regard may be paid to the training of veterinarians for the treatment of the diseases of domestic animals, especially cattle and swine. The representative of the government replied that a scheme for such reorganization had already been prepared and would be gradually carried out. He admitted that the veterinary institutes had heretofore paid almost exclusive attention to horses, but during the last few years cattle and swine diseases had been receiving more and more attention.—*Thierärztliches Centralblatt*, February 15, 1896.

